# Sense of Coherence and Health. The Construction of an Amendment to Antonovsky's Sense of Coherence Scale (SOC II)

**DOI:** 10.1100/tsw.2006.342

**Published:** 2006-06-20

**Authors:** Trine Flensborg-Madsen, Søren Ventegodt, Joav Merrick

**Affiliations:** ^1^ Quality of Life Research Center, Teglgårdstræ de 4-8, DK-1452 Copenhagen K, Denmark; ^2^ Research Clinic for Holistic Medicine, Copenhagen, Denmark; ^3^ Nordic School of Holistic Medicine, Copenhagen, Denmark; ^4^ Scandinavian Foundation for Holistic Medicine, Sandvika, Norway; ^5^ Interuniversity College, Graz, Austria; ^6^ National Institute of Child Health and Human Development, Jerusalem, Israel; ^7^ Center for Disability and Human Development, Faculty of Health Sciences, Ben Gurion University, Beer-Sheva, Israel; ^8^ Office of the Medical Director Division for Mental Retardation Ministry of Social Affairs, Jerusalem, Israel

**Keywords:** Antonovsky, sense of coherence scale, predictability, operationalization, physical health, comprehensibility, manageability, meaningfulness, Denmark

## Abstract

In two previous papers, we concluded that (1) the sense of coherence (SOC) scale developed by Aaron Antonovsky (1923—1994) is unable to prove the association between SOC and the physical health empirically and (2) the SOC scale is unlikely to be a fair materialization of Antonovskys idea and, thus, unlikely to measure SOC correctly. In order to improve the scale, we developed some new questions that we suggest should be incorporated in a new questionnaire and scale (SOC II) derived directly from Antonovsky's idea and the three key explanatory concepts of SOC: comprehensibility, manageability, and meaningfulness. We hope that this new scale will demonstrate a stronger correlation between SOC and physical health.

## INTRODUCTION

The Sense of Coherence Scale developed by Aaron Antonovsky (1923–1994) was intended to measure the *sense of coherence* (SOC), which according to his idea, is of crucial and causal importance to health[1]. In an earlier paper, we concluded that the SOC scale constructed by Antonovsky is highly correlated with psychological aspects[2], but we found that the scale was unable to show strong associations between SOC and physical health as predicted by Antonovsky. We therefore concluded that SOC, as measured by Antonovsky's scale, is a weak predictor of physical health, although a very powerful predictor of psychological measures[3,4,5,6,7,8,9,10,11,12,13], including stress[14,15,16,17,18, 19,20,21] and behavioral aspects[22,23,24,25,26,27]. In an additional paper, we analyzed the idea, the concepts, the theory, and the operationalization behind the SOC scale and concluded that the SOC scale is unlikely to be a fair materialization of Antonovsky's idea and, thus, unlikely to measure SOC correctly[28].

The aim of this paper was to find the questions to be incorporated in a new SOC scale. The scale must be faithful to the original idea of Antonovsky, but we hope that it will demonstrate a stronger correlation between SOC and physical health. We believe that two fundamental problems are present within the prevailing scale. The *first* is the very idea that SOC is merely mental (in the sense of applying to conscious cognition and attitudes), while the questions in the SOC scale are both mental and emotional. In other words, we found that the idea has been contaminated with emotionality in it operationalization[29], which was also proposed in a Canadian study[3]. We recognize three entities of the human being concerning consciousness: the human wholeness (the "soul"), the brain-mind (the "ego" and the "mental" functions), and the body-mind (the "id"). Some researchers argue from a psychological perspective that the "emotional" realm of man is part of the more general "mental" domain. From our perspective, the "mental" functions are dominated by brain function (including somatosensoric modalities), while "emotional" functions are related to the body-mind (which also contains the sensations related to sexuality). The concept of "the human psyche" contains all these dimensions, including the dimensions of consciousness related to the human wholeness (or "soul"). However, we believe that it is the emotionality and not the mentality that determines our physical health and this is the reason why it has not been possible to prove a strong association between SOC and physical health, since the scale mixes the two concepts. The *second* problem is that the scale contains a notion of predictability with the intention that a person scores a high SOC if his life seems predictable, but we believe that is an incorrect assumption.

In this paper, we will only concentrate on the problem of predictability, while the problem of mental and emotional questions will be explored in a succeeding paper[29]. This paper will demonstrate the selection process of the questions for the new scale, that we call SOC scale II. The actual use of the scale will be presented in a subsequent paper using the SOC scale II with a cross-sectional study[30]. The selection of the questions for SOC scale II was based on the same theory as that used by Antonovsky when the original SOC scale was constructed, however, the notion of predictability has been eliminated.

## METHODS

In the construction of a new questionnaire that would best describe the SOC, we based the questions on the theory behind the original questionnaire by Antonovsky with the three dimensions (comprehensibility, manageability, and meaningfulness) and the four personal spheres (inner feelings, immediate interpersonal relations, major activities, and existential issues). The basic questions that we found to belong into these dimensions are presented in Table 1.

The authors propose that the four spheres (from which Antonovsky suggested that it is not possible to narrow the boundaries) can be limited to three spheres: *self*, *life*, and *external world*. This is based on the idea that "self" corresponds to "inner feelings", "life" corresponds to "existential issues", and "external world" corresponds to "major activities". The sphere "interpersonal relations" is not included as a separate sphere, since we believe it is covered by the other three. Interpersonal relation is a functional perspective covered structurally by the other three spheres. The shortened matrix of questions is presented in Table 2.

To concretize the nine basic questions, we used a study where 4,648 Danes aged 31–33 years answered 319 questions[31,32]. Of these questions, 35 were chosen to describe the nine basic questions. Eight collaborators, including two of the authors, divided these 35 questions into 10 categories (the nine abbreviated questions and one fits-nowhere category). The replies from these eight people were analyzed and the 35 questions were divided according to the number of votes they had in each category. The accordance between the replies was impressive and showed a consistent pattern. If a question had the same number of votes for different categories, it was placed according to best consideration. The questions relating to "meaning" were the most consistent as all eight collaborators categorized several of these questions identically. None of the 35 questions were considered to be unacceptable.

**TABLE 1**
**Basic Questions Constructed on the Basis of Antonovsky's Theory**

| | Comprehensibility | Manageability | Meaningfulness |
|---|---|---|---|
| **Inner feelings** | How well do you understand your feelings? | How well do you manage your feelings? | How meaningful do you experience your inner feelings? |
| **Immediate interpersonal relations** | How well do you understand your interpersonal relations? | How well do you manage your interpersonal relations? | How meaningful do you experience your interpersonal relations? |
| **Major activities** | How well do you understand the external world? | How well do you manage your external world? | How meaningful do you experience your external world? |
| **Existential issues** | How well do you understand your existence? | How well do you manage to handle your existence? | How meaningful do you experience your existence? |

**TABLE 2**
**United Basic Questions Based on Table 1**

| | Comprehensibility | Manageability | Meaningfulness |
|---|---|---|---|
| **Self** | How well do you understand your self? | How well do you manage your self? | How meaningful do you experience your self? |
| **Life** | How well do you understand life? | How well do you manage your life? | How meaningful do you experience life? |
| **External world** | How well do you understand your external world? | How well do you manage your external world? | How meaningful do you experience your external world? |

## RESULTS

The final categorization of the questions is shown in Table 3. The questions are the ones that we propose to be integrated in a new SOC scale. This new scale includes 11 comprehensibility questions, 13 manageability questions, and 11 meaningfulness questions.

From the ongoing analysis of the dataset obtained by applying the new scale to the data we have available[31,32], which employs 3- until 7-point scales for each item, we found Cronbach's alpha calculated to be 0.874, which points to a high degree of parallelism in the subjects' response patterns, but not so high that one should discard items that appear well founded on a priori ground. The SOC scale II will be tested in a sequent paper that will reveal how this new scale is highly associated with the physical health[30].

## DISCUSSION

It is worth noticing that even Antonovsky´s famous and frequently used SOC scale, SOC-29, has been under thorough investigation of its validity and reliability since it was first presented in 1987. This indicates that the construction of a valid scale can have many different approaches and opinions. The new scale will be tested in a sequent paper[30]; however, the development of a valid scale should incorporate more aspects of validity and reliability than the ones incorporated in these papers. The construct validity should be investigated using factor analysis, which is an important and powerful method for establishing construct validity. Under optimum conditions, not only one, but also several, longitudinal studies should

**TABLE 3**
**The Categorization of the 35 Questions**

| Basic Questions | Concrete Questions |
|---|---|
| **Comprehension (4 + 3 + 4 = 11 Questions)** | |
| How well do you understand your self? | Do you feel you are open? |
| | Do you feel you are self-confident? |
| | Do you feel you are sincere? |
| | Is there accordance between your thoughts, words, and action? |
| How well do you understand life? | Do you feel you are intuitive? |
| | Do you feel free to choose what your life is going to be like? |
| | Do you feel that fate has treated you badly? |
| How well do you understand your external world? | How good is your current relationship with your present partner? |
| | How good is your current relationship with your friends? |
| | How good is your current relationship with your fellow workers? |
| | How good is your current relationship with your acquaintances? |
| **Manageability (3 + 4 + 6 = 13 Questions)** | |
| How well do you manage your self? | Do you feel you are strong-willed? |
| | Do you feel you are filled with fighting spirit? |
| | Do you accept yourself as you are? |
| How well do you manage your life? | Do you often regret your actions? |
| | Do you easily adjust to new situations? |
| | How satisfied are you with your sex life now? |
| | Do you feel you are competent? |
| How well do you manage your external world? | Do you think that you are involved? |
| | Do you think that you are responsible? |
| | Do you blame others for your unhappiness? |
| | How well are your social needs being fulfilled now? |
| | How well is your need to be useful being fulfilled now? |
| | Do you see problems as a challenge? |
| **Meaningfulness (3 + 5 + 3 = 11)** | |
| How meaningful do you experience your self? | Do you feel you are cheerful? |
| | Do you feel you are a success? |
| | Do you accept yourself as you are? |
| How meaningful do you experience life? | Do you feel your life is a success? |
| | Do you feel that your life has meaning? |
| | How satisfied are you with your life now? |
| | How happy are you now? |
| | How well is your need for an exiting and varied life being fulfilled now? |
| How meaningful do you experience your external world? | Do you feel that your work is meaningful? |
| | Do you feel that you are part of a greater whole? |
| | At present how satisfied are you with the community around you? |

be made in order to investigate the predictive validity of the scale, and also to investigate the responsiveness, i.e., whether the answers are so comparatively stable over time that an observed deviation is likely to represent a prognostic signal and not just instability (noise). Qualitatively, it would be preferable to make interviews in order to disclose the meaning of the questions that are used in the scale. Since some of the questions are very abstract, it would be of great interest to reveal the respondent´s comprehension of the questions and answers. Precisely when a question is of this abstract kind, it will have a potential for deep probing, but only if the words employed are unambiguous. Focus groups would be an advantageous method, as they draw on respondents' attitudes, feelings, beliefs, experiences, and reactions in a way that would not be feasible using other methods, for example, one-to-one interviewing or questionnaires surveys.

## CONCLUSION

In this paper, we have demonstrated our selection process of questions for the new sense of coherence scale (SOC II). The selection is based on the original idea by Antonovsky and the fact that SOC can be divided into comprehensibility; manageability, and meaningfulness. We transformed the four boundaries set up by Antonovsky into self, life, and the external world. The questions in our final questionnaire consist of 11 comprehensibility questions, 13 manageability questions, and 11 meaningfulness questions. The use of this new SOC scale and its association with health will be described in a subsequent paper[30].

## BIOSKETCHES

**Trine Flensborg-Madsen**, **BSc, MSc** (Public Health Science), is currently a research assistant at the Institute of Preventive Medicine, Faculty of Health Sciences, University of Copenhagen, where she is finishing her PhD. Earlier a research assistant at the Quality of Life Research Center in Copenhagen, where she participated in several research projects concerned with holistic health, sense of coherence, and research and follow-up of the Copenhagen Birth Cohort 1959–61. E-mail: tf@imp.hosp.dk Website: http://www.ipm.hosp.dk/person/tfm.htm

**Søren Ventegodt, MD,** is the director of the Nordic School of Holistic Health and Quality of Life Research Center in Copenhagen, Denmark. He is also responsible for a Clinical Research Clinic for Holistic Medicine in Copenhagen and is a popular speaker throughout Scandinavia. He has published numerous scientific or popular articles and a number of books on holistic medicine, quality of life, and quality of working life. His most important scientific contributions are the comprehensive SEQOL questionnaire, the very short QOL5 questionnaire, the integrated QOL theory, the holistic process theory, the life mission theory, and the ongoing Danish Quality of Life Research Survey, 1991–94 in cooperation with the University Hospital of Copenhagen and the late professor of pediatrics, Bengt Zachau-Christiansen, MD, PhD. E-mail: ventegodt@livskvalitet.org. Website: www.livskvalitet.org

**Joav Merrick, MD, MMedSci, DMSc,** is professor of child health and human development affiliated with the Center for Multidisciplinary Research in Aging, Zusman Child Development Center, Division of Pediatrics and Community Health at the Ben Gurion University, Beer-Sheva, Israel; the medical director of the Division for Mental Retardation, Ministry of Social Affairs, Jerusalem; and the founder and director of the National Institute of Child Health and Human Development. Dr. Merrick has numerous publications in the field of child health and human development, rehabilitation, intellectual disability, disability, health, welfare, abuse, advocacy, quality of life, and prevention and received the Peter Sabroe Child Award for outstanding work on behalf of Danish Children in 1985 and the International LEGO-Prize ("The Children's Nobel Prize") for an extraordinary contribution towards improvement in child welfare and well being in 1987. E-mail: jmerrick@internet-zahav.net. Website: www.nichd-israel.com
